# Harnessing pycnidia-forming fungi for eco-friendly nanoparticle production, applications, and limitations

**DOI:** 10.3389/fmicb.2025.1603728

**Published:** 2025-07-31

**Authors:** Mahendra Rai, Beata Zimowska, Sudhir S. Shende, José Milton Elias de Matos, Pramod U. Ingle, Patrycja Golińska, Joanna Trzcińska-Wencel, Aniket K. Gade

**Affiliations:** ^1^Department of Biotechnology, Sant Gadge Baba Amravati University, Amravati, Maharashtra, India; ^2^Department of Chemistry, Federal University of Piaui (UFPI), Teresina, PI, Brazil; ^3^Department of Plant Protection, Institute of Plant Pathology and Mycology, University of Life Sciences in Lublin, Lublin, Poland; ^4^Department of Microbiology, Nicolaus Copernicus University in Toruń, Toruń, Poland; ^5^Department of Biological Sciences and Biotechnology, Institute of Chemical Technology, Mumbai, Maharashtra, India

**Keywords:** agriculture, biosynthesis, mechanism, medicine, nanoparticles, pycnidial fungi

## Abstract

Nanotechnology is pivotal in various fields, including medicine, agriculture, environment, and catalysis. The synthesis of nanomaterials, typically within the 1–100 nm range, can be achieved through physical, chemical, and biological methods. Mycosynthesis, a biological approach, involves using fungi for nanoparticle (NP) synthesis. Several members of the order Pycnidial fungi have recently been reassigned to families such as *Didymellaceae, Mycosphaerellaceae, Botryosphaeriaceae,* and *Diaporthaceae*. Pycnidial fungi, including *Phoma, Phyllosticta, Phomopsis, Macrophomina*, and *Botryosphaeria*, have been reported to mainly synthesize silver and gold NPs, with *Phoma* being the most extensively studied genus. In the present review, keen attention is given to the mechanism of NP synthesis using different members of pycnidial group. The mechanism proceeds through the preparation of a cell-free extract, followed by its treatment with metal precursor salts in the solution. The synthesis of silver or gold NPs occurs *via* the process of reduction of metal ions into respective NPs by various secondary metabolites present in the fungal secretions. This review focuses on the role of pycnidial fungi in synthesizing various NPs, explores the underlying mechanisms, and highlights their significant applications in medicine, the environment, industry, and agriculture. The NPs synthesized from pycnidial fungi are multiplexed for various applications like antimicrobial agents, free radical scavengers, hallmarks for DNA disintegration in cancerous cells, as a potential drug delivery system, as a catalyst, and many more. Although several reports document the role of pycnidial fungi in nanoparticle (NP) synthesis, the precise molecular mechanism underlying NP synthesis still needs to be unraveled before considering their commercial use as microbial factories for biogenic NP production. In addition, the critical challenges in NP synthesis by pycnidial fungi are discussed.

## 1 Introduction

Nanotechnology provides a tool for synthesizing nanoparticles (NPs), which have relevant applications in biology, chemistry, physics, medicine, and agriculture (Alghuthaymi et al., 2022; Li et al., 2023). NPs are considered essential building blocks for nanotechnology. They are the starting points for the background of different nanostructured materials and devices. Because of their extremely small size, dimensions smaller than 100 nm, and high surface area to volume ratio, NPs possess extraordinary physicochemical properties. NPs can be synthesized using three significant methods: chemical, physical, and biological (Rónavári et al., 2021). The chemical method is the most commonly used and traditional approach; however, a downside of this process is the use of chemicals during synthesis, which can sometimes produce materials that may be toxic to cells (Sharma et al., 2022). In the physical methods of synthesis of NPs, sometimes high pressure and temperature are applied, which are harmful (Salem and Fouda, 2021). Consequently, there is a drift toward the synthesis of NPs by using bio-green methods.

The adoption of biological systems for the synthesis of NPs has garnered the attention of nanotechnologists owing to physical and chemical synthesis limitations. It is given advantages over traditional chemical techniques, which can harm the environment. These biological systems include plants, fungi, bacteria, and algae, which follow the principles of green chemistry. Biosynthesis of NPs using biogenic methods is advantageous over chemical and physical methods due to the rapid, clean, simple, non-toxic, inexpensive, and eco-friendly synthesis of NPs (Rai and Golińska, 2021).

The use of fungi for synthesizing NPs as a biological system and applying these synthesized NPs is termed ‘Myconanotechnology’, a term first coined by Rai et al. (2009a). Compared to plants and other microorganisms, fungi are highly efficient in synthesizing various metallic NPs. This is due to their ease of cultivation, rapid growth, secretion of extracellular enzymes, production of a wide range of secondary metabolites, straightforward biomass production, and simple maintenance (Loshchinina et al., 2022). Functional groups such as amine, hydroxyl, thiol, and carboxyl in biomolecules or specific enzymes actively involve redox processes or transform metal ions from precursors to nanoforms. In fact, it is a biochemical process as well, but in the presence of biological matter-biophase (Rai et al., 2023).

As far as mycosynthesized or fungal-mediated synthesis of NPs is considered, there are diverse groups of fungi studied and being explored for their potential in producing biogenic NPs. In the present review, we have focused on one such group of fungi, i.e., pycnidial fungi, being explored for the synthesis of NPs. The pycnidial fungi are characterized by flask-shaped asexual reproductive structures (pycnidium/perithecium), belonging to the kingdom ‘Fungi’ and phylum ‘Ascomycota’ which are prolific producers of secondary metabolites. They liberate the spores through an opening or ostiole in the pycnidium. The majority of representative species belonging to Pycnidial fungi are accommodated in *Didymellaceae, Mycosphaerellaceae, Botryosphaeriacea*, and *Diaporthaceae* (Zimowska, 2022; Ujat and Nakashima, 2023; Dissanayake et al., 2024; Silva-Valderrama et al., 2024). The ability of different pycnidial fungi to reduce the inorganic metals due to the extracellular secretions of reducing metabolites and enzymes was explored as an eco-friendly factor for the synthesis of NPs of different sizes and shapes. This review summarizes the mycosynthesis process, mechanism, stability, and toxicity aspects, and discusses the current applications of NPs synthesized from fungi accommodated in Pycnidial fungi. It emphasizes their broad application prospect and ultimately contributes to a safe and eco-friendly approach to making NPs more biocompatible.

## 2 Mechanism of NP synthesis by pycnidial fungi

Pycnidial fungi are known for forming asexual spores called conidia, which are produced in specialized structures known as pycnidia. These pycnidia are typically flask-shaped or spherical, often with an ostiole. Pycnidial fungi are commonly plant pathogens, saprophytes, or endophytes. Extracellular synthesis of NPs by fungi, in general, and Pycnidial fungi, in particular, offers the advantage of obtaining large quantities of NPs in a relatively pure state and at a rapid rate. Furthermore, the extracellular synthesis of NPs by pycnidial fungi would make the process simpler and easier for downstream processing; fungal broths can be easily filtered by a filter press or similar simple equipment, thus saving considerable investment costs for equipment (Gade et al., 2010).

The extracellular synthesis of NPs by Pycnidial members in general and *Phoma* in particular is a green process (Gade et al., 2013). Understanding the mechanistic aspect of extracellular synthesis of NPs will unravel the biomolecules responsible for the reduction and stabilization of the synthesized NPs. Moreover, elucidation of the mechanism will help to scale up the process of NP synthesis; will allow control over the size, shape, and arrangement of the synthesized NPs; enable an effective strategy for the purification of NPs; increase the stability of the NPs, and can provide substantial information for the functionalization of the NPs.

**Figure 1:**
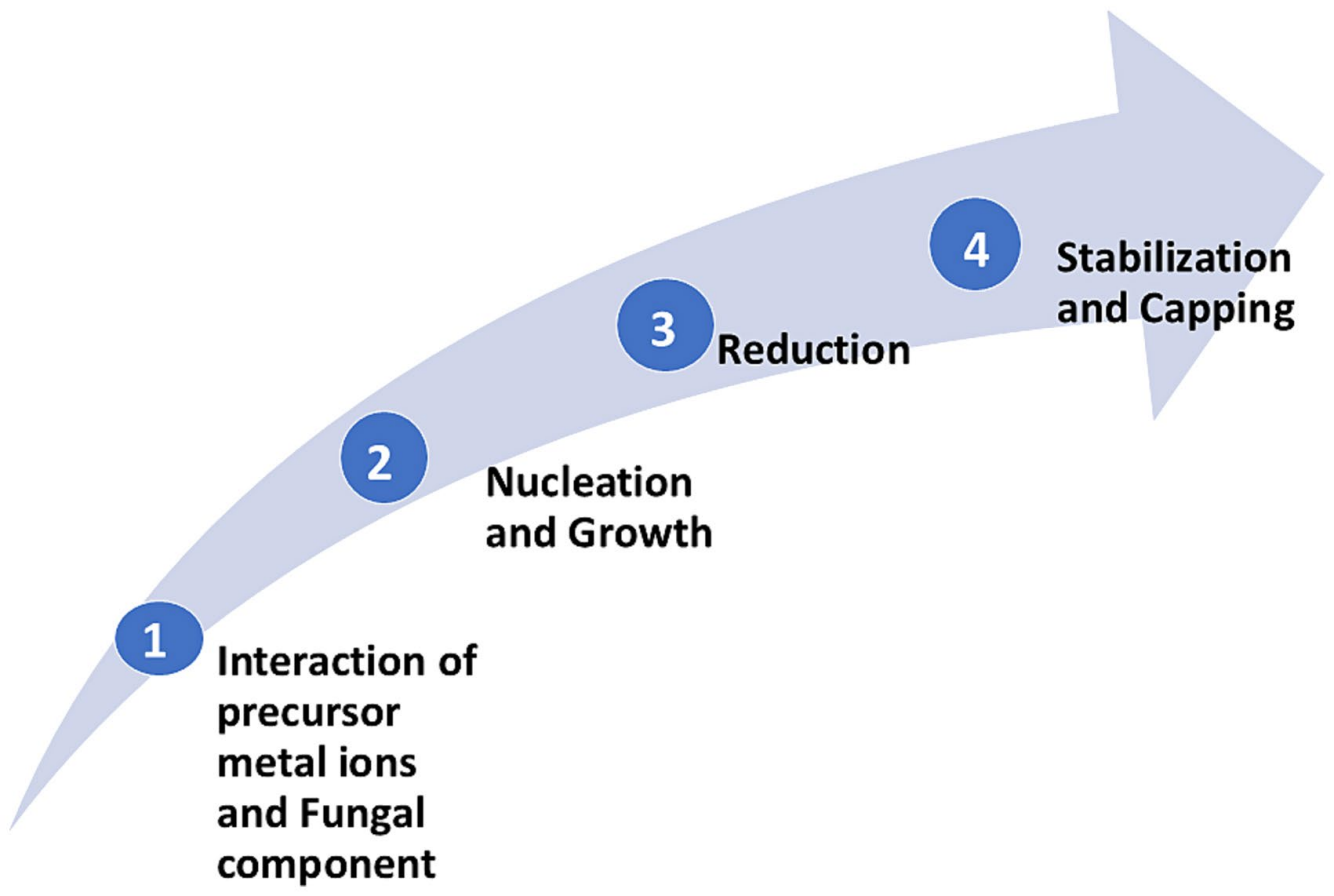
General steps involved in the synthesis of metal NPs by fungi.

The specific mechanisms of NPs synthesis can differ based on the fungal species employed and the type of metal NPs being synthesized. Several factors include precursor metal salt concentration, pH, temperature, light intensity, and the metabolites secreted by the fungus. However, the general steps involved in NP synthesis by the fungi can be summarized below can provide greater insights into understanding the mechanistic aspect of NP synthesis by fungi in general (Figure 1):

1) Metal precursor salt uptake and interaction with fungal components: The synthesis typically begins with the exposure of fungal biomass (intracellular) or fungal secretions (extracellular) to a metal precursor salt. Fungi either absorb the metal ions on the surface or interact with the ions externally *via* biomolecules like proteins (Mukherjee et al., 2008), phenolic compounds, or sugars secreted into the medium (Gade et al., 2013).
2) Nucleation and growth of NPs: Interaction of metal precursor salt with the fungal component leads to the formation of nucleation centers. Nucleation leads to the formation of small clusters that eventually grow to the size of NPs (Gade et al., 2014).
3) Reducing metal precursor ions: Fungal components such as nitrate reductase and anthraquinones, supported by nicotinamide adenine dinucleotide phosphate (NADPH) as a coenzyme (Duran et al., 2005; Sreedharan et al., 2019) or sugars and proteins reduce precursor metal ions. However, Hietzschold et al. (2019) reported that only NADPH acts to reduce silver nitrate to silver NPs.
4) Stabilization and capping of NPs: The biomolecules from the fungal secretions like proteins (Ghaywat et al., 2023), peptides, polysaccharides, and metabolites, cap the synthesized NPs, providing stability, maintaining them in nano-scale and preventing agglomeration. Capping molecules are essential in determining biocompatibility and providing unique physicochemical properties to the synthesized NPs.

The pycnidial fungi comprised genera as *Phoma* (Gade et al., 2022; Ingle et al., 2024), *Phyllosticta* (Manjunatha et al., 2023), *Phomopsis* (Seetharaman et al., 2018), and *Macrophomina* (Spagnoletti et al., 2019), have been reported as efficient fabricators of metal NPs. The understanding of the mechanistic aspects of NPs synthesis by fungi was initially guided by early studies, such as those by Mukherjee et al. (2001). They provided a step-by-step description of the intracellular synthesis of NPs using the fungus *Verticillium* sp. Duran et al. (2005) reported the involvement of the enzyme NADH-dependent reductase in the reduction of silver ions to the formation of silver nanoparticles (AgNPs), which was corroborated by Anilkumar et al. (2007) and Ingle et al. (2008). Further, based on Fourier Transform Infrared Spectroscopy studies, Mukherjee et al. (2008) hypothesized the possible involvement of S–H bond-containing amino acid (cysteine) in forming AgNPs. The cell wall of members of the pycnidial is a dynamic structure that changes and modifies at different stages of the life cycle. It is involved in the absorption of metal ions and plays a vital role in synthesizing metal NPs (Rai et al., 2010). In general, the extracellular synthesis of NPs using fungi is possible either due to the action of reductases, electron shuttle quinones, or both (Duran et al., 2005; Anilkumar et al., 2007; Rai et al., 2011). Among the members of pycnidial, *Phoma sorghina* was studied for the synthesis of silver nanorods, and Gade et al. (2011) proposed a hypothetical three-step mechanism to unravel the mechanistic aspects. They proposed the nucleation, elongation, and termination steps; the nucleation step involves the role of protein in the formation of nucleation centers, and the elongation step involves the photosensitized anthraquinone derivatives acting as an electron shuttle, thereby helping in the elongation of silver nanorod synthesis. The final step is the termination of the silver nanorod synthesis process. The process will be terminated once the anthraquinone molecule cannot transfer the electrons. Whereas for the synthesis of spherical AgNPs by *Phoma glomerata*, the effect of several factors like concentration of metal precursor salt, fungal filtrate, pH, light intensity, and temperature was studied (Gade et al., 2014).

**Figure 2:**
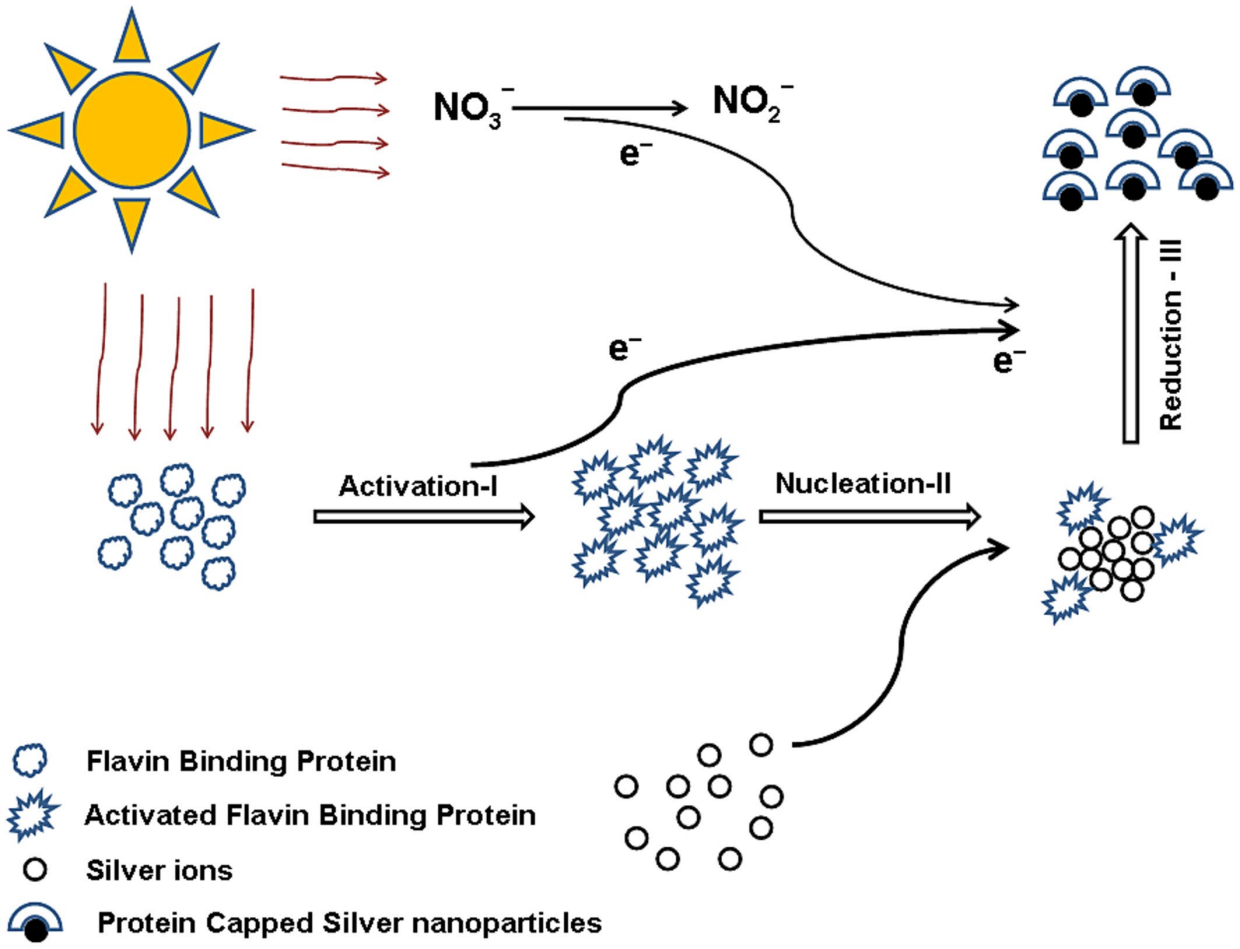
Three-step mechanism of silver NP synthesis by *Phoma glomerata*. Reprinted from “Green synthesis of silver nanoparticles by *Phoma glomerata*”, by Gade et al. (2014), with permission from Elsevier under license 6052381247810.

Based on the outcome, they proposed a three-step mechanism (Figure 2) for synthesizing spherical AgNPs. The three steps are as follows: The first step is an activation of aromatic compounds by photosensitization, where *Phoma glomerata* filtrate is exposed to bright sunlight. The second step is nucleation, where photosensitized aromatic compounds with silver ions initiate the formation of nucleation centers, and the third step includes the reduction of silver ions and the synthesis of spherical AgNPs capped with protein as a stabilizing agent (Gade et al., 2014).

The synthesis of spherical Ag/AgCl NPs by *Macrophomina phaseolina* also indicated the role of nitrate reductase in the synthesis (Spagnoletti et al., 2019). Although most reports are available for AgNPs synthesis, Sreedharan et al. (2019) reported the gold nanoparticle (AuNPs) synthesis by *Macrophomina phaseolina*. They also reported the role of NADPH-dependent reductase in synthesizing AuNPs.

There are several hypothetical mechanisms reported for the synthesis of silver nanorods, spherical AgNPs, and other metal NPs for the members of the pycnidial group. Understanding these mechanisms will enable us to achieve better control over the stability, shape, and size of the synthesized metal NPs. This will lead to the revelation of biochemical pathways for the large-scale production of metal NPs for commercial applications.

## 3 Different members of pycnidial fungi involved in the synthesis of NPs

**Figure 3:**
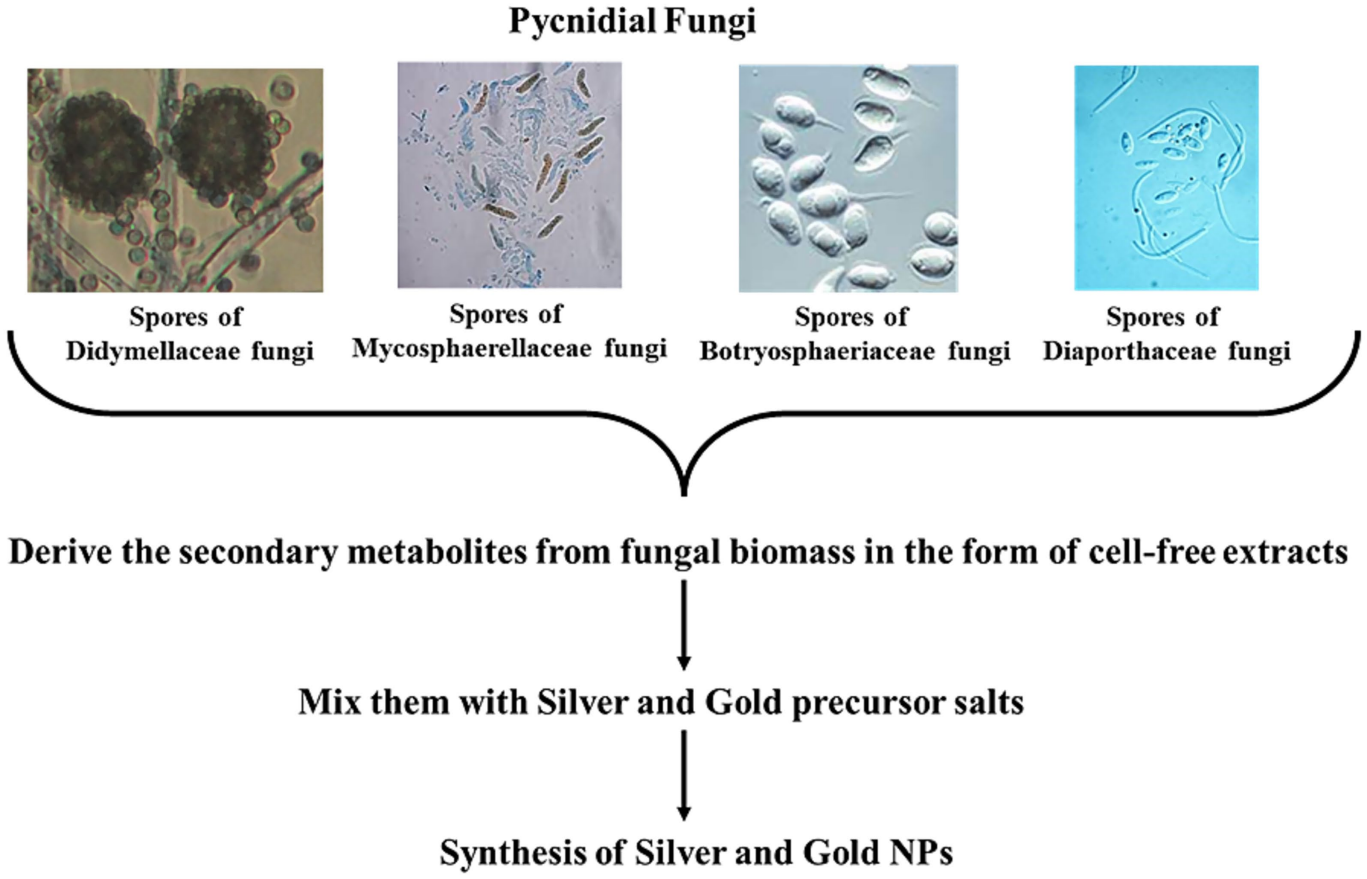
Various pycnidial fungi synthesizing AgNPs and AuNPs.

Various pycnidia-producing fungi can synthesize NPs like gold (AuNPs), silver (AgNPs) (Figure 3), and quantum dots. This represents a significant advancement in green chemistry and offers an eco-friendly alternative to hazardous traditional chemical methods. Various metabolites, enzymes, and other bioactive compounds secreted by the Pycnidial fungi are responsible for the reduction of metal ions into NPs. AuNPs are used in biomedical applications, such as cancer therapy and drug delivery. AgNPs are predominantly antimicrobial, making their use in medical and environmental applications. Catalytic PtNPs from Pycnidial have been reported for their application in industrial processes and therapeutics in cancer treatment (Vahabi et al., 2011; Sreedharan et al., 2019; Zimowska et al., 2024)—fungal members contributing to the Pycnidial fungi group like filamentous fungus *Phomopsis* sp. XP-8 is reported to synthesize composite NPs of Cd_0.5_Zn_0.5_S quantum dots, which are used to functionalize composite mycelium pellet (MCP) for catalytic degradation of visible light (Xu et al., 2021). The following Table 1 summarizes the NPs synthesized by the members of the pycnidial fungi.

**Table 1:** Various NPs synthesized by the members of pycnidial fungi.

Name of fungus	Type of NPs synthesized	Morphology of NPs (shape and size)	Bioactivity	References
*Boeremia exigua var. exigua SYL55*	AgNPs	Hexagonal and triangular with sizes in the range of 10–100 nm.	Antifungal activity against *Boeremia strasseri, Colletotrichum fuscum* and *Sclerotinia sclerotiorum*	Zimowska et al. (2024)
*Botryosphaeria rhodina* MAMB-05	AgNPs	Spherical morphology with a size range of 5–40 nm	Antibacterial against *Staphylococcus epidermidis* and *Escherichia coli*	Muñoz et al. (2024)
*Botryosphaeria rhodina*	myco BR-AgNPs	5–20 nm	Myco BR-AgNPs reduced the growth as well as inhibited the Biofilm formation in ESBL-producing strains of *E. coli.*	Akther et al. (2021)
		5–20 nm	Effective in scavenging free radicals and induced hallmarks of apoptosis including nuclear and DNA fragmentation in lung (A549) cancer cell lines under *in vitro* conditions	Akther et al. (2019)
*Macrophomina phaseolina*	Ag/AgCl-NPs	Spherical morphology with a size range of 5–30 nm	Antibacterial against *Escherichia coli, Bacillus amyloliquefaciens, Pecto-bacterium carotovorum, Pseudomonas protegens pf5, Saccharomyces cerevisiae*	Spagnoletti et al. (2019)
*Macrophomina phaseolina*	AgNPs	Spherical morphology with a size range of 5–40 nm	Bactericidal action against *Escherichia coli* and *Agrobacterium tumefaciens*	Chowdhury et al. (2014)
*Macrophomina phaseolina*	AuNPs	Spherical morphology with a size range 14–16 nm	Potential in improving Drug Delivery System	Sreedharan et al. (2019)
*Phoma* sp.3.2883	AgNPs	71.06 ± 3.46 nm.	Oil industry as an important Catalyst and in the field of human medicine as a bactericide	Chen et al. (2003)
*Phoma glomerata*	AgNPs	60–80 nm	Bactericidal action against *E. coli, Pseudomonas aeruginosa, and Staphylococcus aureus*	Birla et al. (2009)
*Phoma glomerata* MTCC-2210	Silver nanoparticles (SNPs)	Spherical, 66 nm	NA	Gade et al. (2014)
*Phoma gardeniae* ITCC 4554	AgNPs	Spherical and polydisperse NPs within the range of 10–30 nm.	Antimicrobial activity against *Candida albicans, Salmonella choleraesuis, Pseudomonas aeruginosa, Staphylococcus aureus and Escherichia coli.*	Rai et al. (2015b)
*Phoma capsulatum* *Phoma putaminum* *Phoma citri*	AgNPs	10–80 nm 5–80 nm 5–90 nm Shape-Spherical and irregular	Antimicrobial activity against *Aspergillus niger, Candida albicans, Salmonella choleraesuis, Pseudomonas aeruginosa, Staphylococcus aureus and Escherichia coli.*	Rai et al. (2015a)
*Phoma* sp. MN995524	AgNPs	12.7 nm, Spherical	Antibacterial activity against *Escherichia coli, Pseudomonas aeruginosa, and Klebsiella pneumoniae*	Sonbol et al. (2022)
*Phoma sorghina* MTCC 2906	AgNPs	120–160 nm with a width range from 30–40 nm	NA	Gade et al. (2011)
*Phoma arachidicola, Phoma betae, Phoma exigua* var. *exigua*, *Phoma glomerata*, *Phoma fimeti*, *Phoma herbarum*, *Phoma destructiva*, *Phoma exigua*, *Phoma medicaginis, Phoma multirostrata*, *Phoma* sp.*, Phoma sorghina*	AgNPs	Polydispersed spherical AgNPs, with the average size of the particle ranging between 58 and 84 nm.	NA	Gade et al. (2013)
*Phoma* sp	AuNPs	Spherical with sizes in the range of 10–100 nm	Antifungal and Antibacterial activity against phytopathogens including *Rhizoctonia solani* and *Xanthomonas oryzae* pv. *oryzae*	Soltani Nejad et al. (2022)
*Phoma exigua* NCIM1237	AuNPs	20–50 nm	Antioxidant properties	Chakravarty et al. (2015)
*Phoma glomerata* MTCC 2210	AuNPs	78 nm	Energy technology, Catalyst in methanol fuel cell	Kuralkar et al. (2015)
*Phomopsis liquidambaris* SA1	AgNPs	average size of 18.7 nm, shape-Spherical and polydispersed	Antimicrobial against *Vibrio cholera, Salmonella typhi, Micrococcus luteus, Proteus mirabilis, Escherichia coli, Klebsiella pneumonia, Shigella flexneri, Pseudomonas putida* and Mosquitocidal against *Aedes aegypti* and *Culex quinquefasciatus*	Seetharaman et al. (2018)
*Phomopsis* sp. GFPA2	AgNPs	10–100 nm, crystals	Antibacterial against *Staphylococcus aureus* ATCC 25623, *Bacillus cereus* ATCC 6633, *Pseudomonas aeroginosa* ATCC 27853 and *Escherichia coli*	Sutjaritvorakul and Chutipaijit (2018)
*Phomopsis* sp. XP-8	AuNPs, Cd_0.5_Zn_0.5_S quantum dots	NA	Nano-Photocatalyst application in degradation of visible light	Xu et al. (2021)
*Phyllosticta owaniana*: KUMBMDBT-32	AgNPs	65.81 nm, Spherical shape	Antibacterial activity against *Pseudomonas aeruginosa* Antifungal activity against *Candida albicans*	Manjunatha et al. (2023)
*Phyllosticta* sp.	AgNPs	70–112 nm in cell filtrate, 62.02–82.11 nm in the fungal mat	Antibacterial activity against *Pseudomonas aeruginosa, Staphylococcus aureus*	Vardhana et al. (2018)
*Phyllosticta capitalensis*	AgNPs	20–50 nm	Antioxidant, Antidiabetic and Antimicrobial potential against: *Listeria monocytogenes*, *Staphylococcus aureus*, *Escherichia coli* and, *Salmonella typhimurium*.:	Andrade et al. (2024)
*Septoria apii*	AgNPs	5–30 nm, Spherical shape		Huang et al. (2013)

## 4 Applications of NPs synthesized by pycnidial fungi

Pycnidial fungi, a group of fungi, have gathered interest for their use in the synthesis of NPs and their potential use in various fields because of their distinct properties (Boroumand et al., 2015). Pycnidial fungi are reported to produce many secondary metabolites and bioactive compounds, making them a promising candidate for NP green synthesis (Loshchinina et al., 2022). Biological systems are used to reduce the metal ions in an eco-friendly green approach, forming NPs with characteristic size, shape, and functionalities (Kaur et al., 2022; Altammar, 2023; Kazemi et al., 2023; Malik et al., 2024). This includes genera such as *Phoma*, *Phyllosticta*, *Phomopsis*, and *Macrophomina*. Various NPs have been synthesized using these fungi and are reported to have applications in different fields. Here, the NPs synthesized using the members of pycnidial fungi are discussed.

### 4.1 Biomedical applications

NPs synthesized by the members of Pycnidial fungi can be promisingly used for various applications, especially in medicine and pharmaceuticals. The Pycnidial-mediated NPs could be functionalized to target specific tissues and cells, making them ideal drug delivery systems (Joseph et al., 2023; Yusuf et al., 2023). The drug loading capacity of these NPs is increased due to their small size and large specific surface area, which improves their therapeutic efficacy and minimizes the probability of side effects. In addition, the secondary metabolites from the fungi can confer various properties, like anticancer, antimicrobial, and anti-inflammatory, to these NPs (Gomes et al., 2021; Huang et al., 2024). Another exciting application of pycnidial fungi-mediated NPs is in biosensing and diagnostics. The NPs can be used in conjugation with the enzymes, antibodies, and DNA, enabling their binding to the target site specifically (Malhotra and Ali, 2018; Doria et al., 2012). This property helps design sensitive and specific nano-based biosensors for disease detection, pathogen identification, and eradication of environmental pollutants (Mokhtarzadeh et al., 2017; Valenzuela-Amaro et al., 2023). Many of the NPs functionalized with the specific antibodies can be used for the detection of biomarkers and can be precise and rapid diagnostic tools (Wang and Wang, 2014; Combes et al., 2021).

Akther et al. (2019) have studied the anticancer property of AgNPs synthesized from *Botryosphaeria rhodina* an endophyte from *Catharanthus roseus* plant. They demonstrated the potential anticancer activity of these mycogenic AgNPs using A549 cancer cells as a model system through apoptosis (Akther et al., 2019). Later in 2021, they reported the reduction of growth and inhibition of biofilm formation in ESBL-producing strains of *Escherichia coli* as a model organism. Both these reports the multimodal action mechanism of *B. rhodina* mediated AgNPs, indicating their potential use in medicinal industries as an alternative antimicrobial or anticancer agent (Akther et al., 2021).

For example, *Phoma* spp. may be terrestrial or marine and produce bioactive compounds having properties like antimicrobial, herbicidal, phytotoxic, and anti-cancer (Rai et al., 2009b; Rai et al., 2018; Rai et al., 2022a). Chen et al. (2003) and Gade et al. (2013) have studied different species of *Phoma* for their ability to synthesize AgNPs. The studies have confirmed the great potential of *Phoma* spp. in metallic NPs synthesis (Edmundson et al., 2014). *P. exigua* is another species that produces AgNPs (Karmakar et al., 2010). Endophytic *Phyllosticta* sp. mediated AgNPs demonstrated antimicrobial and larvicidal activity, indicating their potential use in the medical field (Seetharaman et al., 2018; Vardhana et al., 2018). AgNPs synthesized by a strain of *Phomopsis* sp. (strain GFPA2) showed antibacterial activity against human pathogenic bacteria such as *E. coli* (ATCC 27853), *Pseudomonas aeruginosa* (ATCC 27853), *Bacillus cereus* (ATCC 6633), and *Staphyloccocus aureus* (ATCC 25623) which indicates their use as an alternative nano-based antimicrobial agent in the field of medicine (Sutjaritvorakul and Chutipaijit, 2018).

### 4.2 Environmental applications

The field of environmental remediation can be revolutionized using the NPs mediated by Pycnidial fungi (Guerra et al., 2018; Roy et al., 2021). The NPs can be used to adsorb and degrade the pollutants from soil and water. Various heavy metals and organic contaminants can be removed from the water bodies via reduction, adsorption, and catalytic degradation (Ningthoujam et al., 2022; Ali et al., 2023). The biological nature of NPs ensures the eco-friendliness of application to avoid the use of hazardous chemicals involved in the remediation processes and other environmental applications (Kazemi et al., 2023). *P. exigua* NCIM1237 mediated AuNPs have shown to have anti-oxidant activities and are proved using assays like DPPH (2,2-diphenyl-1-picrylhydrazyl) quenching assay and hydrogen peroxide assay (Chakravarty et al., 2015).

### 4.3 Industrial applications

Industrially important NPs have been reported to be precious and used in various applications like catalysis, and electrophoresis, (Dikshit et al., 2021). Catalysis is a significant application that studies the chemical reactions involving NPs derived from members of the Pycnidial fungi (Navalón and García, 2016). These NPs have unique surface properties and active sites, enhancing their reaction rates and selectivity (Khan et al., 2017). For example, AuNPs derived from Pycnidial fungi have been explored for their role in organic reactions, such as oxidation and reduction processes. The biological mode of synthesis of NPs involves the biomolecules as a capping agent that prevents agglomeration and stabilizes the NPs, which helps in enhancing catalytic efficiency (Mikhailova, 2021; Roy et al., 2022; Santhosh et al., 2022). *P. glomerata-*mediated AuNPs are reported as catalysts for industrial application by Kuralkar et al. (2015). The removal of elemental contaminants like selenium (Se) and tellurium (Te) from the environmental samples by converting them into their oxidized NP form is another application of *P. glomerata* species (Liang et al., 2019). This elemental remediation process helps remove heavy metal contaminants, aiding their role in sustainable environmental remediation. In another study, Liang and colleagues reported the biomass enrichment of *P. glomerata* when exposed to Se/Te ores. This helps purify Se and Te’s volcanogenic sulfide deposits from natural reservoirs (Liang et al., 2020).

### 4.4 Agricultural applications

Various mycosynthesized NPs have been explored and applied in agriculture for their distinctive functionalities (Sudheer et al., 2022; Šebesta et al., 2022a). This includes nano fertilizers, nano pesticides, and nano herbicides, which are sprayed, dusted, or applied as stable suspension as a soil supplement (Mittal et al., 2020; Nongbet et al., 2022). *Phoma*, along with *Penicillium, Fusarium*, and *Aspergillus*, are among the important fungi that play a key role in plant growth promotion, resulting in improved agricultural productivity (Hossain et al., 2017; Rai et al., 2021; Mgadi et al., 2024). For instance, a popular *Phoma* sp., *P. herbarium*, was used for the fabrication of copper oxide (CuO) NPs (83 nm) and was evaluated against fungal pathogens isolated from ginger, i.e., *P. aphanidermatum* and *F. oxysporum*. Significant inhibition of fungal pathogens was recorded, indicating the effective use of *Phoma* sp. for the fabrication of nano-fungicide for agricultural purposes (Ghaywat et al., 2023).

## 5 Key issues in biosynthesis using pycnidial fungi

The biogenic green synthesis of NPs using microorganisms, such as those from the Pycnidial fungi, presents unique opportunities due to its cost-effectiveness, environmentally friendly nature, and potential for producing diverse bioactive NPs. However, several challenges must be addressed to realize its full potential. These challenges primarily include (i) scalability and reproducibility of the biosynthesis process and (ii) the stability and toxicity of the NPs, which are discussed below.

### 5.1 Scalability and reproducibility of the biosynthesis process

The intrinsic variability in biological systems is a significant challenge for the biogenic green synthesis of NPs using pycnidial fungi. Variations in environmental conditions can affect the NP size, shape, and yield, complicating the scalability of the process (Gade et al., 2008; Narayanan and Sakthivel, 2010; Shende et al., 2017; Gade et al., 2022). The metabolic activities of microorganisms may vary, impacting NP production, which can be further exacerbated in large-scale operations.

Scaling up from lab-scale to industrial-scale production requires careful optimization of growth conditions, such as temperature, pH, carbon and nitrogen sources, and aeration. These factors must be precisely controlled to maintain consistency in NP synthesis (Rai et al., 2011; Shende et al., 2017). Differences in mixing efficiency and mass transfer in more extensive systems can lead to reproducibility issues, making it challenging to produce uniform NPs consistently.

Moreover, the cost involved in the synthesis process is a critical factor. The biogenic synthesis process at larger scales may become more expensive, particularly concerning the substrates and growth media required. Developing cost-effective methods for culturing Pycnidial fungi and extracting NPs is essential to ensure economic viability (Ahmad et al., 2015). Additionally, the availability of resources, including large volumes of nutrients, poses another challenge that could impact the sustainability and scalability of the process.

### 5.2 Stability and toxicity of NPs formed from pycnidial fungi

NPs produced through biological synthesis often face stability issues. Factors such as particle size, surface charge, and residual biological molecules can lead to agglomeration or changes in particle properties over time, affecting their application in various fields (Das and Marsili, 2010; Behravan et al., 2019). Environmental conditions like temperature and humidity further influence these NPs stability and shelf life.

The Pycnidial fungal members are widely known for their ability to biosynthesize NPs through their secondary metabolites by offering an eco-friendly alternative to chemical methods (Elhamouly et al., 2022). However, their toxicity and environmental impacts are understudied concerning their long-term scenario. The biocatalytic activity of these NPs is influenced by fungal metabolites, their particle size, exposure duration, and concentration (Šebesta et al., 2022b; Yadav et al., 2023). These bioactivities can result in cytotoxicity, DNA damage in cells, and oxidative stress, which is a function of their intrinsic properties. Prolonged exposure may cause an unintended harmful effect on non-targeted organisms, including aquatic life and soil microbiota, which has remained unexplored over a period of time. Potential bioaccumulation and trophic transfer raise concerns about their interactions with food chains and ecological persistence (Kaur et al., 2019; Ameen et al., 2021; Juan et al., 2021; Xuan et al., 2023).

The fungal members of the pycnidial group have the potential to synthesize metal NPs like AgNPs and AuNPs, their critical aspects such as electrostatic stability, bioavailability and cytotoxicity remain largely unexplored. Comprehensive investigation of these parameters is essential to determine their suitability for biomedical and agricultural applications which are the attributes of their stability and bioavailability. There are some biogenic AgNPs and AuNPs which have been evaluated for these types of important properties. The stability of the biogenic NPs is a function of nucleophilic ions in their capping layer which is derived from the biological counterpart during synthesis. These ions that are adsorbed on the surface prevent aggregation, resulting in smaller-sized stable NPs in colloidal form (Gurunathan et al., 2009). Stabilization can also be accomplished by mycelial proteins and enzymes from fungal filtrates, where negative carboxyl groups can impart electrostatic stability (Husseiny et al., 2015). Cytotoxicity of these NPs is a function of size, shape and surface morphology. Akther et al. (2019) studied the cytotoxicity of mycogenic AgNPs from *Botryosphaeria rhodina* at concentrations of 5, 10, 20, 40, 60, 80, and 100 μg/mL by MTT assay against A549 cells. They assessed the cytotoxic effect of AgNPs based upon the damage to genetic material and induction of apoptosis in the test eukaryotic cells. Thus, it can be predicted that the NPs derived from other members of pycnidial fungi would behave in a similar fashion.

Concerning the regulatory standpoint, the unavailability of standardized guidelines for fungal-mediated NPs presents challenges in evaluating their safety for biomedical and environmental applications. Unlike chemically synthesized NPs, which have established risk assessment protocols, biological NPs derived from pycnidial fungi require in-depth studies on their degradation, toxicity thresholds, and long-term environmental persistence. The regulatory gaps concerning the way fungi-derived NPs are made, the materials used, and how they interact biologically are different from what existing guidelines cover. Since NPs fabricated by fungi through intra-or extracellular biosynthesis pathways demonstrated the involvement of organic coatings (e.g., fungal proteins and other metabolites) that change the stability and toxicity profiles (Adeleke et al., 2024). In addition, they exhibit different size distribution and aggregation behaviors from chemically synthesized counterparts and may also retain bioactive fungal components, such as enzymes that influence the environmental fate and risk benefits (Adeleke et al., 2024). No OECD procedures exist to characterize these biogenically mediated properties, notwithstanding their crucial impact on risk assessment results (Adeleke et al., 2024). Although OECD guidelines direct physico-chemical characterization like particle size distribution, dissolution kinetics, and others [OECD (Organisation for Economic Co-operation and Development), 2022], they are unable to address the fungal-specific synthesis byproducts such as mycotoxins, secondary metabolites, the biological perseverance of fungal templates in NMs structure (Adeleke et al., 2024), and dynamic alteration in NPs’ properties during fungal-mediated reduction (Baun et al., 2017). Besides this, there are some critical knowledge gaps highlighted in literature that involve (i) fate modeling: traditional environmental fate models lack parameters for fungal-mediated NP modification processes (Baun et al., 2017); (ii) dosimetry: existing metrics (mass/volume) are insufficient for fungi-derived NPs with heterogeneous organic–inorganic interfaces (Adeleke et al., 2024); (iii) tiered testing strategies: there is no OECD-approved method for determining when fungal synthesis pathways require supplementary testing (Kah et al., 2021). Framework modifications to track both the NPs and their fungal-derived components through environmental compartments (Baun et al., 2017; Kah et al., 2021). These are the gaps that persist despite increasing application of fungal nanotechnology in different fields like medicine and agriculture (Adeleke et al., 2024), underscoring the need for the OECD to expand its guidelines beyond chemically fabricated NPs. The reliance of the present framework on conventional chemical risk paradigms [OECD (Organisation for Economic Co-operation and Development), 2022] creates regulatory blind spots for biogenically synthesized NPs, potentially underestimating risks from fungal-derived NPs interactions.

Regulatory bodies must address the need for robust toxicity profiling and risk assessment frameworks before widespread commercialization (Irshad et al., 2024; Malik et al., 2024). Additionally, ethical and legal considerations, such as biosafety approvals and environmental monitoring, must be integrated into NP development policies. Future research should focus on multi-generational toxicity studies, real-world exposure models, and green disposal strategies to ensure that pycnidial fungus-mediated NPs align with sustainable and safe nanotechnology practices.

**Figure 4:**
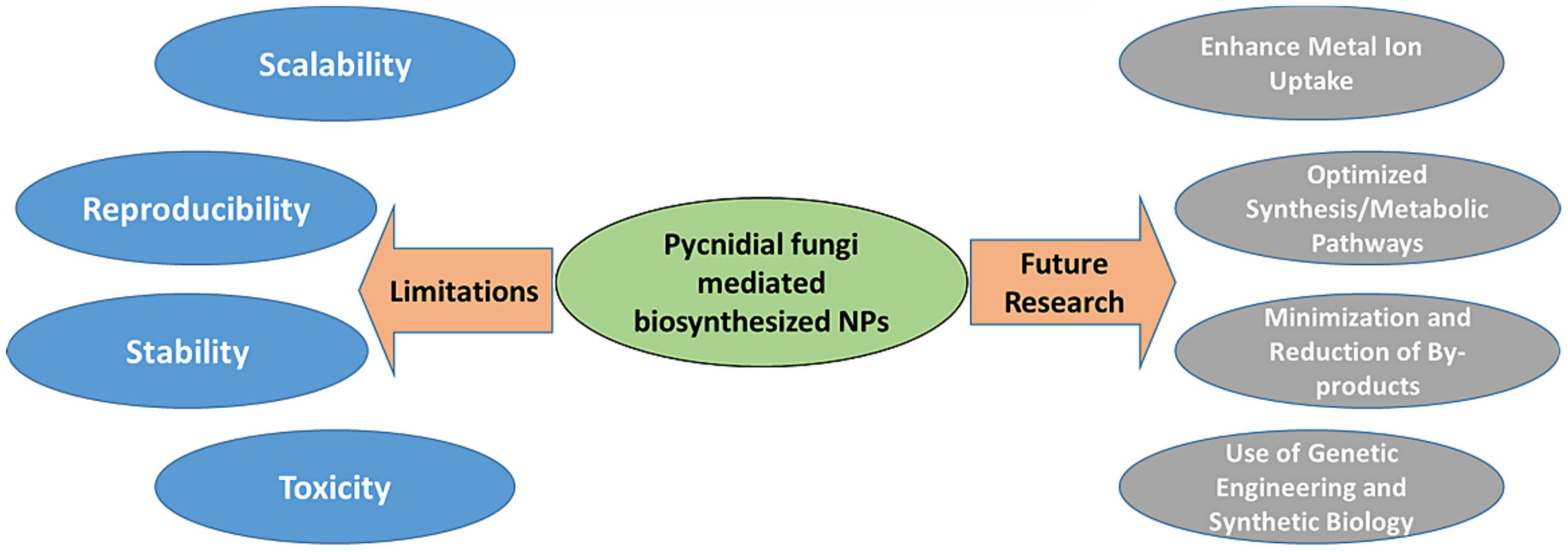
Limitations and future research in development on pycnidial fungus mediated NPs.

Assessing the potential toxicity of NPs prepared from Pycnidial fungi is crucial. Due to their small size and high surface area, these NPs might interact differently with biological systems, posing potential health and environmental risks (Oberdörster et al., 2005; Khan et al., 2017). These NPs surface characteristics and composition, including any residual biological molecules from the synthesis process, can contribute to their toxicity. A comprehensive toxicity assessment is necessary to understand their impact and establish safe handling protocols. Figure 4 represents some of the major limitations and future research aspects to be explored in the research and development on pycnidial fungus-mediated NPs.

## 6 Future outlook

Future research should focus on addressing variability and improving reproducibility in the biosynthesis of NPs. Developing standardized protocols is essential, including optimizing growth conditions and implementing consistent quality control measures (Narayanan and Sakthivel, 2010; Rai et al., 2015a; Rai et al., 2022b). Automating the culturing and monitoring the synthesis processes could help in scaling up production while minimizing human error, leading to more reliable outcomes.

A key area of research that requires development in metabolic engineering to enhance the efficiency of Pycnidial fungi in NP synthesis. This can be achieved through several strategies:

- ***Enhanced metal ion uptake:*** Engineering the metal ion uptake mechanisms in Pycnidial fungi can increase the availability of metal ions for NP synthesis. For instance, modifying membrane transporters to improve the uptake of specific metal ions can enhance the biosynthesis process (Behravan et al., 2019).
- ***Optimized metabolic pathways:*** Optimizing metabolic pathways to boost the production of reducing agents, such as organic acids or proteins, can improve the efficiency of NP synthesis. Metabolic engineering strategies can redirect metabolic fluxes toward these pathways, thereby increasing the overall yield of NPs (Das and Marsili, 2010).
- ***Reduction of by-products:*** Genetic modifications that minimize the production of by-products, which may interfere with NP synthesis, can be advantageous. For example, engineering pathways to reduce the generation of unwanted organic compounds can lead to purer and more consistent NP synthesis (Singh et al., 2016).
- ***Genetic engineering and synthetic biology***: Advances in genetic engineering can create Pycnidial fungi strains with enhanced NP synthesis capabilities, including higher yields and more uniform particles. Synthetic biology approaches can further enable the design of microbial factories tailored for large-scale production, potentially reducing costs and improving efficiency (Gade et al., 2008, 2022; Singh et al., 2016).

Genetic engineering can also enhance NP production by modifying metal reduction and NP formation genes. For example, upregulating genes encoding reductase enzymes, crucial in reducing metal ions to NPs, can increase yield and control size and shape (Narayanan and Sakthivel, 2010). Overexpressing enzymes such as nitrate or sulfite reductase can enhance the reduction process, leading to more efficient NP synthesis (Gade et al., 2008, 2022).

Addressing the stability and toxicity of NPs is another critical issue. This could be achieved through surface modification techniques, such as coating NPs with biocompatible polymers or adding stabilizing agents (Sharma et al., 2009; Das and Marsili, 2010; Shende et al., 2017). Functionalizing the surfaces of biogenic NPs with specific molecules can also help reduce toxicity by preventing unintended biological interactions and extending their shelf-life whose safety can be evaluated through development of risk assessment frameworks (Shende et al., 2017). This includes assessing their behavior in biological and environmental systems and understanding their long-term effects (Qu et al., 2013). Establishing regulatory guidelines will ensure the safe use of these NPs, mitigating potential health and environmental impacts.

Promoting the recovery and recycling of NPs can reduce their environmental impact and support a circular economy. Developing methods for the efficient separation and reuse of NPs in different treatment processes is essential (Wiesenthal et al., 2011; Hansen et al., 2022). For NPs that cannot be recycled, implementing environmentally friendly disposal practices will help protect ecosystems and prevent pollution. Finally, exploring other fungal species within the Pycnidial fungi is urgently needed to identify potential candidates for diverse NP synthesis.

## 7 Approaches for mitigating these limitations

Scalability is one of the major limitations in NPs production in large quantities. In the case of plant systems, the secondary metabolites the plants are exposed to certain biotic and/or abiotic stress, which may trigger a series of signal transduction pathways involving the synthesis of desired metabolites and are extracted for synthesis of extracellular synthesis. This is commonly known as the elicitation of metabolite technique. Various physical and chemical factors are used for triggering these pathways, such as the mitogen-activated protein kinase (MAPK) pathway, production of reactive nitrogen and oxygen species, and activation of NADPH oxidases. The technique can be followed for the fungal metabolite production as well (Martínez-Chávez et al., 2024).

On the other hand, the metabolite engineering approach, a technique of improved cellular activity by manipulating metabolism, can also be used for enhanced production of secondary metabolites. Here, the flow of carbon is guided toward the desired metabolic pathway, resulting in the blockage of intermediate steps and the synthesis of alternative products like enzymes in the system. These can be used as potential reducing equivalents for NPs synthesis (Martínez-Chávez et al., 2024). Metabolic engineering is reported for the production of bacteria-mediated AgNPs using a sustainable approach of random or site-directed mutagenesis to overcome the cons of high cost and NPs toxicity. As the desired metabolites can be produced in excess quantities, the synthesis of NPs is ultimately enhanced through these strain improvement protocols. Fungal strains can also be improved to obtain enhanced production at larger scales (Mitra et al., 2018). Metabolic engineering is also used in enhancing the production of fatty acids in yeast systems (Ullah et al., 2021). Fungi are well-known for their secretory metabolite pool, which is mainly composed of various proteins and enzymes. They can serve as a capping and stabilization agent during NPs synthesis (Rami et al., 2024). Protein engineering is another platform for manipulating the protein profile of an organism through protein performance optimization, selectivity, solvent and thermal stability, and substrate/product interactions. Cellular transformations and enzyme expressions can be controlled to derive desired stabilizing and capping materials from microbial sources like bacteria and fungi (Dorcheh and Vahabi, 2016).

Thus, it is evident and important to optimize the metabolite synthesis intra-and extracellular levels to get enhanced production, which will ease their extraction and stabilization of NPs during synthesis.

## 8 Conclusion

Pycnidial fungi, a novel group of fungi, has gained attention for its ability to biosynthesize various NPs, including AuNPs, AgNPs, and nanocomposites, which have diverse applications. Fungi such as *Phoma*, *Phyllosticta*, *Septoria*, *Phomopsis*, and *Macrophomina* offer an eco-friendly alternative for NP synthesis through the action of intra-and extracellular enzymes and secondary metabolites. These molecules facilitate the reduction of metal ions and stabilize the synthesized NPs via nucleation. This biological approach is advantageous over conventional chemical and physical methods, which often require harsh conditions like high temperatures, pressure, and toxic reagents. Enzymes such as reductases, along with fungal metabolites (proteins, lipids, and carbohydrates), play a crucial role in reducing metal ions and capping NP surfaces, resulting in NPs with varying shapes and sizes. Most NPs synthesized through this process exhibit antimicrobial properties, making them suitable for therapeutic applications such as antimicrobials, drug delivery, cancer therapy, and biosensing technologies. NPs synthesized from Pycnidial fungi also hold the potential for developing wound dressings, coatings, antibacterial textiles, and more. Their photocatalytic properties further suggest their use in environmental remediation, such as water purification and air filtration.

Pycnidial fungi stand as a novel members for the small-scale and commercial production of metal-based NPs due to their huge pool of secondary metabolites. These assist in harboring specific biomedical or catalytic activity to the prepared NPs, which decides their respective application. Whereas, the precise mechanism lying behind the Pycnidia-mediated NPs synthesis needs to be thoroughly explored to control, monitor and understand the *in situ* progress of the reaction to determine the type of enzymes, transporters and stabilizing agents involved. The ever-growing applicability and despite these promising applications, the biochemical mechanisms underlying NP synthesis by Pycnidial fungi warrant further studies. Future research focused on improving process control, optimizing fungal strains, and elucidating the mechanisms of mycosynthesis will expand their potential applications. Further, studies based upon designing the risk assessment framework aligned with the recent updated guidelines from OECD [OECD (Organisation for Economic Co-operation and Development), 2022] upholding manufactured nanomaterials and their assessment of safety measures and potential risk factors involved would be necessary for the commercial application of Pycnidia-mediated NPs in various fields such as biomedicine, environment, industry, and agriculture.
